# Determinants of childbirth care quality along the care continuum in limited resource settings: A structural equation modeling analysis of cross-sectional data from Burkina Faso and Côte d’Ivoire

**DOI:** 10.1186/s12884-021-04328-w

**Published:** 2021-12-29

**Authors:** Tieba Millogo, Raïssa Kadidiatou Kourouma, Bertrand Ivlabéhiré Méda, Marie Laurette Agbre-Yace, Abdul Dosso, Maurice W. E. Yaméogo, Seni Kouanda

**Affiliations:** 1 African Institute of Public Health (AIPH), 12 BP 199, Ouagadougou, Burkina Faso; 2 Institut de Recherche en Sciences de la Santé, Ouagadougou, Burkina Faso; 3 Institut National de Santé Publique (INSP-Côte d’Ivoire), Abidjan, Côte d’Ivoire; 4 Cellule de Recherche en Santé de la Reproduction, Abidjan, Côte d’Ivoire

**Keywords:** Continuum of childbirth, Quality of care, Burkina Faso, Côte d’Ivoire

## Abstract

**Introduction:**

Despite the important increase in in-facility births, perinatal mortality rates have remained high and slow to decrease in many developing countries. This situation is attributed to poor childbirth care quality. The reason why women delivering in health facilities do not always receive care of an adequate standard is unclear. We assessed the determinants of childbirth care quality along the care continuum by means of different approaches.

**Methods:**

A health facility-based cross-sectional study with a direct observation of health care workers’ practices while caring for mother–newborn pairs was carried out in Burkina Faso and Côte d’Ivoire. The performance of a set of essential best practices (EBPs) was assessed in each birth event at the admission, prepushing and immediate postpartum stages. A quality score, in the form of the additive sum of EBPs effectively delivered, was computed for each stage. We used negative binomial regression models and a structural equation modeling analysis to assess the determinants of care quality at each stage and the relationships of the quality delivered at the different stages, respectively.

**Results:**

A total of 532 and 627 mother–newborn pairs were evaluated in Burkina Faso and Côte d’Ivoire, respectively. In both countries, delivery care quality varied significantly at all stages between health districts. Predelivery care quality was consistently higher in referral hospitals than in primary health care facilities (incident rate ratio (IRR) = 1.02, *p* < 0.05, and IRR = 1.10, *p* < 0.05, respectively, for Burkina Faso and Côte d’Ivoire). Quality at admission was poorer among nurses than among midwives in Burkina Faso (IRR = 0.81, *p* < 0.001). Quality at the admission and predelivery stages was positively correlated with immediate postpartum care quality (β = 0.48, *p* < 0.001, and β = 0.29, *p* < 0.001, respectively).

**Conclusion:**

Quality improvement strategies must target both providers and health facilities, and different inputs are needed depending on the stage in the care continuum.

## Background

Timely utilization of health facilities for childbirth services has been strongly advocated throughout the past two decades in low- and middle-income countries (LMICs) [1, 2], where maternal and newborn death tolls remain unacceptably high despite recent improvements. Nonaccess or delays in access to needed interventions that are proven and well recognized as effective for preventing or adequately managing obstetrics complications were identified as major contributing factors to poor childbirth outcomes [3, 4]. The promotion of institutional childbirths as a strategy to reduce poor maternal and newborn childbirth outcomes relies on the postulate that such interventions are readily available in health facilities and effective conditional on the utilization of health care services. Health facility utilization alone, however, has proved insufficient [5–7]. The quality of the care received by the mother–newborn dyad during the course of childbirth in health facilities is also crucial; indeed, as facility utilization is rising, it is gradually being recognized as a missing link in the path toward ending preventable maternal and child deaths in LMICs where the quality of care is often reported to be suboptimal [8, 9]. In-facility morbidity and mortality outcomes depend heavily on the quality of health care received by service users, as it conditions the effective delivery of interventions that meet their health needs [10]. The quality of childbirth health care services not only improves the medical outcomes of childbirth but also is a key factor for improving and maintaining an optimal level of service utilization. Indeed, previous studies in sub-Saharan Africa (SSA) have reported that women’s future decisions on whether to use health facilities for delivery depend on their previous use experience [11, 12]. Those not satisfied with the services received were less likely to sustain their service utilization [13]. Because access alone is no longer sufficient, access to quality essential health services by 2030 is one of the targets (SDG target 3.8) under the United Nations’ (UN) Sustainable Development Goal 3 (SDG 3), which promotes measures to ensure healthy lives and well-being for all at all ages [14]. Care quality is complex [15] and encompasses various dimensions, including the care structure, process and outcomes, each requiring equal attention in efforts to improve quality [16]. Structural aspects of quality and health care outcomes, including fatalities and user satisfaction, are frequently used to assess the quality of maternity care in SSA [17, 18]. The quality of the care process may, however, more adequately pinpoint the gap between the desired and actual care received and provide more information on areas needing improvement [19]. There is no consensus in the literature on how and which indicators to use to measure the quality of the delivery process and labor care [20]. Different approaches, including records reviews [21], user interviews [22], and less frequently direct observations of care practices, have been used [23]. Record reviews are considered less reliable because they may fail to accurately document all actions performed during the care process, and disagreements often arise between experts after reviewing the same records [24]. Identifying and assessing the performance of actions from user accounts may also be challenging [25]. Direct observations of care practices by an expert, although challenging to implement, are the gold standard for evaluation [26]. The tools or quality indicators to be used are another issue that must be addressed [27]. There is a plethora of indicators, making it almost impossible to claim that an evaluation is complete [21]. The validity and reliability of many indicators used remain questionable, as they are very often evaluated by means of expert opinions [28]. The use of empirically validated indicators is rather infrequent but would improve quality measurement by providing a set of comprehensive indicators and thus solve issues related to the proliferation and inherent measurement errors of indicators [29].

Previous studies using validated indicators for routine delivery care reported suboptimal care quality, with an important fraction of women delivering in health facilities failing to receive easy-to-perform and low-cost interventions [23, 30]. While resource scarcity is a known obstacle to the delivery of high-cost interventions, it does not always fully explain the lack of provision of low-cost yet effective interventions. There is a need for further understanding of the factors that favor or hamper the delivery of low-cost and easy-to-perform interventions during childbirth to inform quality improvement strategies. Based on a list of comprehensive quality indicators derived from a validated quality index, delivery care quality has been assessed and reported elsewhere [30]. Here, we report on the determinants of care quality by using typical regression methods along with a structural equation modeling analysis. Among its other advantages, the latter method allows us to account for the specificities of the indicators used to measure care quality at each point instead of treating them all as equal in weight and to further explore the relationships along the care continuum, where quality at a later stage may depend on the quality received at previous stages.

## Methods

### Data

The study was conducted in Burkina Faso and Côte d’Ivoire. The first country is a low-income country (LIC), while the latter is ranked as a lower middle-income country (LMIC) by the World Bank. Both countries have made important progress in shifting deliveries from home to health facilities in recent years, with 66.3 and 73.6% of all deliveries taking place in health facilities in Burkina Faso and Côte d’Ivoire, respectively. However, these improvements in health facility utilization for childbirth were not followed by the expected improvement in childbirth outcomes. Improvements to maternal mortality ratios (MMRs) have stalled, with MMRs estimated to be as high as 320 and 617 per 100,000 live births for Burkina Faso and Côte d’Ivoire, respectively. Neonatal mortality rates are also high, with 28 and 38 deaths per 1000 in Burkina Faso and Côte d’Ivoire, respectively [31, 32].

The study was conducted in selected health regions (one in each country) where the research teams were located and could easily conduct the field activities. Both regions (Central-North in Burkina Faso and Agnéby-Tiassa-Mé in Côte d’Ivoire) have their capital cities located approximately 100 km from the respective country capital cities and have similar healthcare delivery systems, with one top regional hospital followed by district hospitals (six in Burkina and seven in Côte d’Ivoire), each serving a health district comprised of several primary healthcare facilities (PHCFs).

A facility-based cross-sectional study consisting of direct observation of childbirth practices was conducted in a sample of PHCFs and referral hospitals (regional or district hospitals) in each country. We first randomly selected five health districts. The district hospitals of the selected health districts along with the regional hospital were systematically included in the study in each country. In the catchment area of each selected health district, up to 15 eligible PHCFs were randomly selected to be included in the study. Eligible PHCFs were those with a minimum average caseload of 1.5 childbirths per day, as reported in the health records of the same period in the previous year (the last quarter of 2017). Details on the sample size calculation are reported elsewhere [30]. In these health facilities, data collectors with a medical background (medical students and midwives), who had undergone a two-day training including study-room role plays and field testing of the study tools, were installed in the delivery room to directly observe and document healthcare providers’ adherence to a set of signal functions while caring for pregnant women and their future newborns at four critical points: admission, before the woman started pushing (predelivery), immediately postpartum (soon after delivery up to ≤1 h), and before discharge from the health facility. Data from the first three pause points are reported in the current study. Health care providers who were in their qualifying internship period in the health facility were excluded from the observations. Pregnant women aged < 18 years and those admitted at an advanced stage of labor (third stage) with frequent (≤5 min apart) and intense uterine contractions that would prevent them from providing valid informed consent were also excluded from the study. Data collection took place from 12 November to 20 December 2018 and lasted for up to 5 days in each health facility. Observations were conducted every day of the week and at any time without discontinuation, provided an eligible pregnant woman was present in the delivery room. A data collector could observe all or some of the pause points for each birth event. However, all data pertaining to the same critical juncture were collected by the same data collector. Thus, once a data collector had initiated observation for a given pause point, he or she could complete the shift only after having filled in all items pertaining to that pause point.

### Measures

#### Quality indicators

Delivery care quality was assessed using a set of 30 signal functions and tracers derived from the WHO Safe Childbirth Checklist (WHO-SCC) [33] complemented by a review of a list of validated and recommended signal functions for SSA [29]. A total of twelve (12) quality indicators (yes/no) were assessed in each childbirth event at the admission and initial examination stage: the provider washes his or her hands before examination, the provider wears sterile gloves before vaginal examination, the provider asks for historic vaginal bleeding during pregnancy, the provider asks for historic vision blurring, the provider checks the woman’s HIV status or performs an HIV test where missing, the provider measures the woman’s blood pressure, the provider takes the woman’s pulse, the provider measures the woman’s temperature, the provider uses a partograph to monitor the progress of labor where indicated, the provider assesses the woman for need for referral for a higher level of care, the provider encourages the companion/husband to assist with the childbirth and the provider advises the husband/companion to call in case a complication occurs. Eight similar yes/no indicators were assessed at the predelivery stage (before pushing): sterile gloves are readily available, soap and clean water are readily available, the provider prepares a prefilled syringe with uterotonics for active management of the third stage of labor (AMTSL), a clean towel is readily available, sterile blade or scissors are readily available, the provider prepares a face mask for neonatal resuscitation, the provider has a suction device readily available, and an assistant birth attendant is identified and ready to intervene if needed.

At the third pause-point, the performance of the following ten (10) signal functions (yes/no) was assessed: the provider correctly clamps or ties the cord, the provider immediately dries the newborn with a clean towel, the provider assesses the mother for vaginal and perineal lacerations, the provider assesses the placenta for completeness, the provider takes the mother’s vital signs (blood pressure, pulse) within 15 min of the delivery, the provider palpates the uterus within 15 min of the delivery, the mother is encouraged to initiate breastfeeding within 1 h of the delivery, skin-to-skin contact is initiated within 1 h of the delivery and the provider advises the companion and/or woman to call in case of complications.

#### Determinants of care quality

Quality determinants were categorized into three groups: user, provider and health facility characteristics. Data were collected on the following user characteristics: maternal age, maternal educational level (none, primary and secondary/tertiary), number of pregnancies over the woman’s entire reproductive life and attendance of at least four antenatal care (ANC) visits during the pregnancy. The cut-off of four ANC visits was used because this was in line with official guidelines in both countries at the time of the data collection. Provider characteristics included the qualification of the health care worker attending the childbirth event (midwife, nurse or other auxiliary staff), provider age and gender, and total years of working experience. Other auxiliary staff included nurse and midwife assistants and were found only in Burkina Faso. The following health facility characteristics were included in the study: facility type (primary health care facility versus referral hospital), availability of clean running water, availability of electricity, health district to which it belongs, and residential location (rural versus urban).

### Statistical analyses

We performed three series of analyses. In the descriptive statistics, we used proportions and means plus standard deviations to describe qualitative and quantitative variables, respectively. The univariate analysis included the use of the chi squared test or independent t-test to compare proportions and means. In the second phase, we carried out negative binomial regressions to identify the determinants of delivery care quality for each of the three pause points and each country. Covariates selection was informed by literature review [23, 34–36]. The dependent variable for the negative binomial regression models was a quality score that resulted from the additive combination of the dichotomized quality indicators pertaining to each stage. This quality score had a maximum of 12, 8 and 10 for the admission, predelivery and soon after delivery phases, respectively. The minimum score was 0 for all stages. For a birth event to be included in the analysis for a given pause point, all relevant quality indicators had to be assessed for that specific pause point. Complete data were available for 1069 (92.9%), 1100 (94.9%) and 1064 (91.9%) birth events at admission, predelivery and soon after delivery, respectively. A negative binomial regression model was preferred to Poisson regression because of its robustness to overdispersion. The analyses performed at this stage were based on the complete case analysis. A missing data category was, however, included in the models of Côte d’Ivoire for the variable maternal education, which had 7.3% missingness. In the third set of analyses, we used the structural equation modeling (SEM) technique to identify, in addition to the determinants of care quality, the interrelationships between care quality in the different phases along the care continuum. Our model for the continuum of childbirth care includes the three critical points in childbirth care: initial examination (admission), before the woman starts pushing for delivery (before pushing) and immediately postpartum (soon after birth). The quality at each of these points can be influenced by both health facility and provider characteristics. The quality at a given stage is also susceptible to influence from the quality at early stages. The SEM technique allows assessment of the latent variables corresponding to the quality of care at each stage by means of the observed dichotomized indicators (the measurement component of the model) and effects of quality at an earlier phase on quality at a later phase (the structural component of the model). A country group comparison allowing the structural path coefficients to vary did not show a significant difference between the two models, and we finally retained a unique SEM for both countries. The root mean square error of approximation (RMSEA) and comparative fit index were used to check the overall fit of the final SEM. The RMSEA of 0.06 indicated a close to perfect goodness of fit. Traditionally, an RMSEA below 0.08 shows a satisfactory fit, and an RMSEA below 0.05 is a perfect fit. Most of the observed indicators were significant at *p* < 0.05 [37]. All analyses were performed using Stata 15.1, and a significance level of *p* < 0.05 was used. Because data were clustered within health facilities, we used robust standard errors.

## Results

### Background characteristics

The study was conducted in 142 health facilities (73 in Burkina Faso) of which 12 were referral hospitals. Childbirth care practices of 529 health care providers were observed in both countries. A total of 532 and 627 pregnant women were included in the admission phase of the study in Burkina Faso and Côte d’Ivoire, respectively. Once included in the study (pause point I), a woman and thereafter her newborn were observed through the subsequent pause points (II and III) unless they were referred to another health facility or transferred to a different service. The mean age of women was 25 years in Burkina and 26.5 years in Côte d’Ivoire (*p* < 0.001). The vast majority (87.63%) of the childbirth events in Côte d’Ivoire and just more than half (57.99%) in Burkina Faso (*p* < 0.001) were attended by midwives. The provider’s mean years of working experience was greater in Côte d’Ivoire than in Burkina Faso. Detailed information on the characteristics of the women, providers and health facilities is presented in Table 1.

**Table 1:** Backgrounds characteristics of study populations

	Burkina Faso n (%) *N* = 532	Côte d’Ivoire n (%) *N* = 627	***P***-value (Chi2 or t-test)
**User’s characteristics**			
***Maternal age: mean (sd)***	25.08 (6.08)	26.47 (6.33)	<0.001
***Maternal education***			
*None*	432 (85.48)	309 (53.26)	<0.001
*Primary*	58 (8.78)	181 (26.26)	
*Secondary/tertiary*	42 (5.74)	98 (13.16)	
*Missing*	–	39 (7.31)	
***Number of ever pregnancies: mean (sd)***	1.10 (0.84)	1.17 (0.75)	0.147
***Four ANC visits-Yes***	309 (57.45)	328 (54.30)	0.402
**Distribution of the birth events observed according to health providers’ characteristics**			
***Qualification***			
*Midwives*	347 (57.99)	575 (87.63)	<0.001
*Nurses*	56 (13.36)	52 (12.37)	
*Other auxiliary staffs*	129 (28.65)	–	
***Gender***			
*Male*	154 (32.59)	42 (10.21)	<0.001
*Female*	378 (67.41)	585 (89.79)	
***Provider’s age: mean (sd)***	33.64 (4.55)	37.57 (4.82)	<0.001
***Years of working experience***	4.32 (4.18)	6.32 (5.09)	<0.001
**Distribution of the birth events observed according to health facilities’ characteristics**			
**Urban location of the health facility**	141 (9.97)	284 (29.14)	<0.001
***Rural location of the health facility***	391 (90.03)	343 (70.86)	<0.001
***Facility has clean water (pipe)-Yes***	291 (46.64)	420 (58.96)	0.001
***Facility has electricity (including solar power)- Yes***	504 (92.62)	621 (96,89)	0.052
***Health districts***			
*Kaya/Agboville*	101 (20.59)	118 (21.86)	
*Boulsa/Akoupé*	109 (20.59)	135 (10.99)	
*Kongoussi/Tiassalé*	118 (22.05)	121 (21.86)	
*Barsalogho/ALEPE*	102 (17.66)	120 (21.86)	
*Tougouri/Adzopé*	102 (19.12)	133 (23.42)	

### Negative binomial regression

#### Burkina Faso

The qualification of the provider along with his/her age and gender showed a significant association in multivariable analysis with care quality at admission. After we adjusted for other factors, nurses and auxiliary staff had lower quality rates (incident rate ratio (IRR) = 0.81–95% CI (0.72–0.90), *p* < 0.001, and IRR = 0.88–995% CI (0.80–0.96), *p* < 0.01, respectively) at admission than midwives. Quality at admission was also lower in female providers (IRR = 0.89–995% CI (0.82–0.96), *p* < 0.01) and greater as the provider’s age increased (IRR = 1.01–95% CI (1.01–1.02), *p* < 0.01). Quality at predelivery showed an association with facility characteristics. Referral hospitals had higher-quality predelivery care (IRR = 1.02–995% CI (1.01–1.18), *p* < 0.05), and rural health facilities had lower care quality at this phase (IRR = 0.92–95% CI (0.86–0.99), *p* < 0.05). Immediate postpartum care quality was positively associated with provider age (IRR = 1.01–9 95% CI (1.00–1.02), *p* < 0.01).

#### Côte d’Ivoire

There were important differences in care quality at both stages between health districts. Referral hospitals had higher-quality predelivery care (IRR = 1.10–995% CI (1.01–1.20), *p* < 0.05) and lower-quality immediate postpartum care (IRR = 0.84 (0.77–0.93)) than primary health care facilities. Providers’ years of working experience were positively correlated with immediate postpartum care quality (IRR = 1.01–9 95% CI (1.00–1.02), *p* < 0.05). The crude and adjusted incidence rate ratios of care quality at all three stages in both Burkina Faso and Côte d’Ivoire are presented in Table 2.

**Table 2:** Crude and adjusted incidence rate ratios for quality of care at admission, pre-delivery and immediate post-partum and per country

	Crude Incidence rate ratio (IRR, 95% confidence interval)			Adjusted Incidence rate ratio (IRR, 95% confidence interval)		
**Burkina Faso**						
**Variables**	Admission (*n* = 532)	Before pushing	Soon after birth	Admission	Before pushing	Soon after birth
**User characteristics**						
Maternal age	0.99 (0.99–1.01)	0.99 (0.99–1.00)	0.99 (0.99–1.00)	0.99 (0.98–1.00)	0.99 (0.99–1.01)	0.99 (0.99–1.00)
Maternal education (ref = None)						
*Primary*	1.06 (0.94–1.19)	0.93 (0.86–1.01)	0.97 (0.90–1.05)	1.04 (0.93–1.16)	0.93 (0.86–1.02)	0.95 (0.89–1.02)
*Secondary/tertiary*	1.07 (0.96–1.18)	1.02 (0.93–1.13)	1.01 (0.91–1.11)	0.92 (0.81–1.04)	0.98 (0.89–1.09)	0.95 (0.86–1.04)
Number of ever pregnancies	0.98 (0.94–1.03)	0.99 (0.97–1.02)	0.97 (0.94–0.99)	1.00 (0.94–1.07)	0.99 (0.96–1.03)	0.99 (0.94–1.03)
Four ANC visits (ref = No)	1.02 (0.95–1.10)	1.01 (0.96–1.06)	1.01 (0.96–1.06)	1.00 (0.94–1.07)	1.02 (0.98–1.06)	1.09 (0.97–1.05)
**Provider characteristics**						
Qualification (ref = midwives)						
*Nurses*	0.91 (0.81–1.03)	1.04 (0.97–1.11)	0.98 (0.92–1.04)	0.81 (0.72–0.90) ***	0.99 (0.93–1.05)	0.96 (0.91–1.03)
*Other auxiliary staffs*	0.94 (0.86–1.03)	0.97 (0.92–1.02)	0.97 (0.91–1.03)	0.88 (0.80–0.96) **	0.96 (0.91–0.99)*	0.95 (0.89–1.00)
Gender (ref = Male)	0.94 (0.87–1.07)	0.93 (0.90–0.98)**	0.96 (0.92–1.01)	0.89 (0.82–0.96) **	0.99 (0.94–1.04)	0.98 (0.92–1.03)
Provider’s age	1.02 (1.01–1.03)***	1.01 (1.00–1.01)**	1.01 (0.99–1.01)	1.01 (1.01–1.02) **	1.00 (0.99–1.01)	1.01 (1.00–1.02)**
Years of working experience	1.02 (1.01–1.02)***	1.01 (1.00–1.01)*	1.01 (1.00–1.01)***	1.00 (0.99–1.01)	1.00 (0.99–1.01)	–
**Facility characteristics**						
Referral hospital (ref = PHCF)	1.11 (1.04–1.19)**	1.15 (1.09–1.20)***	0.98 (0.93–1.04)	1.07 (0.96–1.20)	1.02 (1.01–1.18)*	0.96 (0.90–1.03)
Rural location (ref = urban)	0.88 (0.81–0.95)**	0.91 (0.86–0.97)**	0.90 (0.86–0.95)***	1.02 (0.90–1.16)	0.92 (0.86–0.99)*	0.97 (0.91–1.02)
Full staff on duty at childbirth (ref = No)	1.06 (0.99–1.15)	1.01 (0.96–1.06)	1.02 (0.97–1.08)	1.02 (0.95–1.10)	1.02 (0.97–1.07)	1.02 (0.98–1.08)
Facility has clean water (pipe)	1.12 (1.05–1.21)**	0.93 (0.89–0.97)**	0.99 (0.95–1.04)	1.03 (0.95–1.11)	0.95 (0.92–0.99)*	0.99 (0.94–1.03)
Health districts (ref = Kaya)						
*Boulsa*	0.93 (0.84–1.01)	1.13 (1.06–1.20)***	0.94 (0.88–1.01)	0.94 (0.85–1.04)	1.14 (1.07–1.21) ***	0.96 (0.89–1.04)
*Kongoussi*	0.68 (0.61–0.76)***	1.06 (1.01–1.12)*	0.98 (0.94–1.03)	0.66 (0.59–0.75) ***	1.06 (0.99–1.12)	0.99 (0.93–1.05)
*Barsalogho*	0.76 (0.69–0.84)***	1.06 (0.99–1.14)	0.94 (0.89–0.99)*	0.75 (0.66–0.85) ***	1.07 (1.00–1.14) *	0.95 (0.88–1.02)
*Tougouri*	0.74 (0.68–0.81)***	0.83 (0.77–0.88)***	0.79 (0.74–0.84)***	0.75 (0.68–0.83) ***	0.85 (0.79–0.91) ***	0.81 (0.75–0.87)***
**Côte d’Ivoire**						
**Variables**	Admission (*n* = 627)	Before pushing	Soon after birth	Admission	Before pushing	Soon after birth
**User characteristics**						
Maternal age	0.99 (0.99–1.00)	0.99 (0.99–1.00)	0.99 (0.99–1.00)	0.99 (0.99–1.00)	1.00 (0.99–1.01)	0.99 (0.99–1.00)
Maternal education (ref = None)						
*Primary*	0.96 (0.88–1.04)	0.98 (0.92–1.04)	0.94 (0.89–0.99)*	1.00 (0.94–1.08)	0.98 (0.92–1.04)	0.96 (0.91–1.01)
*Secondary/tertiary*	0.96 (0.87–1.06)	1.04 (0.95–1.13)	0.88 (0.82–0.96)*	1.02 (0.92–1.15)	1.10 (1.01–1.21)*	0.96 (0.88–1.04)
Missing	0.93 (0.62–1.47)	1.15 (1.07–1.22)	1.10 (0.96–1.25)	0.68 (0.53–0.86)	1.15 (0.96–1.37)	1.12 (0.87–143)
Number of ever pregnancies	0.99 (0.94–1.05)	0.99 (0.97–1.02)	1.01 (0.98–1.05)	1.02 (0.97–1.07)	0.95 (0.90–0.99)*	1.05 (1.00–1.09)*
Four ANC visits (ref = No)	1.03 (0.95–1.12)	0.99 (0.94–1.04)	1.02 (0.97–1.07)	1.03 (0.96–1.10)	0.95 (0.86–1.01)	1.03 (0.98–1.08)
**Provider characteristics**						
Qualification (ref = midwives)						
*Nurses*	0.91 (0.79–1.05)	0.92 (0.85–0.99)*	0.89 (0.82–0.97)**	0.93 (0.77–1.12)	0.94 (0.80–1.11)	0.92 (0.78–1.08)
*Other auxiliary staffs*						
Gender Female (ref = Male)	1.08 (0.91–1.27)	1.12 (1.03–1.22)*	1.14 (1.03–1.28)**	1.04 (0.83–1.32)	1.18 (0.93–1.48)	1.19 (0.97–1.47)
Provider’s age	0.99 (0.98–1.00)	1.01 (1.00–1.01)*	1.00 (0.99–1.01)	1.01 (0.99–1.02)	1.00 (0.99–1.01)	1.00 (0.99–1.01)
Years of working experience	0.99 (0.98–1.01)	1.01 (1.00–1.01)**	1.00 (0.99–1.01)	1.00 (0.99–1.01)	1.00 (0.99–1.01)	1.01 (1.00–1.02)*
**Facility characteristics**						
Referral hospital (ref = PHCF)	0.82 (0.74–0.91)***	1.10 (1.04_1.15)***	0.88 (0.82–0.94)***	0.77 (0.68–0.87)	1.10 (1.01–1.20) *	0.84 (0.77–0.93)***
Rural location (ref = urban)	0.96 (0.87–1.05)	0.99 (0.94–1.04)	0.98 (0.94–1.03)	1.02 (0.96–1.09)	1.05 (0.99–1.12)	0.99 (0.94–1.06)
Full staff on duty at childbirth (ref = No)	1.04 (0.94–1.14)	0.97 (0.92–1.03)	0.98 (0.94–1.03)	1.02 (0.95–1.09)	1.00 (0.93–1.07)	1.01 (0.94–1.08)
Facility has clean water (pipe)	1.05 (0.96–1.15)	1.01 (0.95–1.06)	0.96 (0.91–1.01)	1.05 (0.98–1.13)	1.06 (0.99–1.13)	1.03 (0.97–1.09)
Health districts (ref = Adzopé)						
*Akoupé*	0.85 (0.77–0.93)***	1.12 (1.03–1.22)**	0.95 (0.88–1.03)	0.86 (0.78–0.95)**	1.17 (1.07–1.28) **	0.95 (0.87–1.03)
*Alepé*	1.24 (1.14–1.35)***	1.04 (0.94–1.15)	1.15 (1.07–1.24)***	1.30 (1.18–1.43) ***	1.11 (1.00–1.23)*	1.19 (1.11–1.29)***
*Tiassalé*	1.03 (0.88–1.21)	1.17 (1.07–1.28)***	1.29 (1.19–1.41)***	1.06 (0.84–1.34)	1.31 (1.15–1.49) ***	1.37 (1.20–1.55)***
*Agboville*	0.88 (0.82–0.96)**	1.21 (1.11–1.31)***	1.01 (1.01–1.15)*	0.85 (0.78–0.94) **	1.23 (1.13.-1.33) ***	1.01 (0.92–1.10)

*p < 0.05 **p < 0.01. ***p < 0.001

### Structural equation modeling analysis

**Fig. 1:**
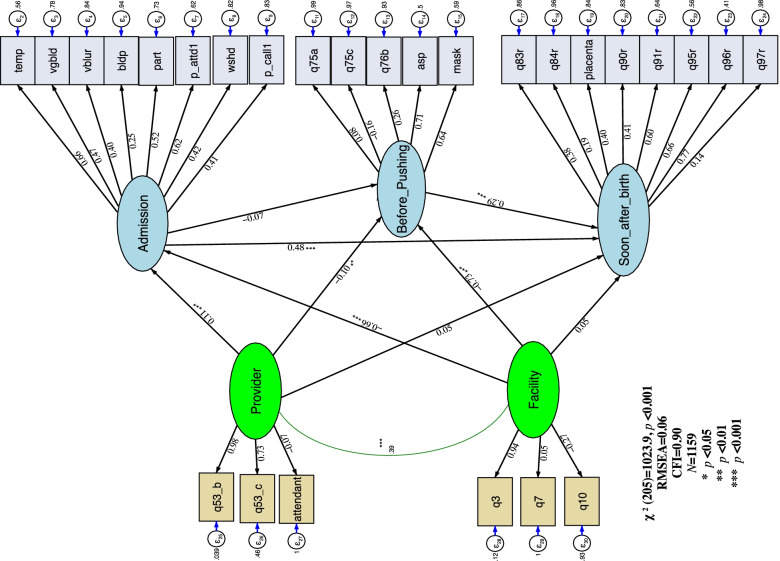
This figure depicts the structural equation model for the analysis of the determinants of childbirth care quality along the continuum. The measurement component of the model has two parts: the latent endogenous quality variables (Admission, Before pushing, Soon after birth) and their respective observed indicator variables and the latent exogenous variables: provider and facility variables along with their observed indicator variables. The structural component is composed of the relationships between the latent exogenous and latent endogenous variables. The standardized coefficients are presented on the path

Figure 1 presents the standardized parameter estimates for care quality at the three phases. Two items had strong loadings on care quality at the admission stage (i.e., their coefficients were highly significant): the provider encourages the companion to assist the delivery (*β* = 0.62; *p* < 0.001) and the provider measures the woman’s temperature (*β* = 0.66; *p* < 0.001). The indicators that strongly defined predelivery care quality were the ready availability of a suction device (*β* = 0.71; *p* < 0.001) and the availability of a face mask for neonatal resuscitation (*β* = 0.64; *p* < 0.001). The two indicators with the highest loadings on care quality at the immediate postpartum stage were early initiation of skin-to-skin contact (*β* = 0.77; *p* < 0.001) and early initiation of breastfeeding (*β* = 0.66; *p* < 0.001). The results of the measurement part of the SEM are presented in Table 3.

**Table 3:** standardized parameter estimates of the measurement indicators of the SEM model

Codes	Explanations (labels)	Standardized parameter estimate (Se)	t-value
***Admission***	***Quality of initial assessment***		
Wshd	Washes his/her hands before examination	0.42 (0.04)	11.56 ***
Vgbld	Asks for vaginal bleedings	0.47 (0.03)	13.64 ***
Vblur	Asks for vision blurred	0.40 (0.04)	11.22 ***
bldp	Measures blood pressure	0.25 (0.04)	6.85***
part	Uses partograph where indicated	0.52 (0.03)	15.68 ***
Temp	Takes temperature	0.66 (0.03)	18.69 ***
P_attd1	Companion encouraged to assist to childbirth	0.62 (0.05)	13.06 ***
P_call1	Informs companion/woman to call if complications	0.41 (0.03)	13.50 ***
***Before_pushing***	***Before pushing or before caesarian section***		
q75a	Sterile gloves readily available	0.08 (0.04)	2.38 **
q75c	Soap and clean water readily available	−0.16 (0.03)	−4.87 ***
q76b	Sterile blade or scissor readily available	0.26 (0.04)	7.52 ***
mask	Face mask readily available	0.64 (0.04)	14.82 ***
asp	Suction device readily available	0.71 (0.05)	14.94 ***
***Soon_after_birth***	***Immediate postpartum (within one hour)***		
q83r	Clamps or ties cord correctly	0.38 (0.03)	11.63 ***
q84r	Immediately dries the baby with clean towel	0.19 (0.04)	5.16 ***
placenta	Assesses for placenta completeness	0.40 (0.03)	13.09 ***
q90r	Takes mother vital signs within 15 min after	0.41 (0.06)	7.09 ***
q91r	Palpates uterus within 15 min after birth	0.60 (0.06)	10.83 ***
q95r	Initiates breastfeeding within 1 h	0.66 (0.02)	26.16 ***
q96r	Skin-to-skin contact initiated within 1 h	0.77 (0.02)	31.25 ***
q97r	Informs companion/woman to call if complications	0.14 (0.03)	4.62 ***
***Provider***	***Provider’s indicators***		
q53_b	Age of the provider	0.98 (0.00)	1144.20 ***
q53_c	Years of working experience of the provider	0.73 (0.01)	49.03 ***
attendant	Qualification of the provider	−0.07 (0.03)	−2.30 *
***Facility***	***Health facility indicators***		
q3	Health district of the facility	0.94 (0.002)	375.30 ***
q7	The health facility has electricity	0.05 (0.03)	1.52
q10	Hands washing devices/facilities available	−0.27 (0.03)	−9.50 ***

*p < 0.05. **p < 0.01 ***p < 0.001

The results of the structural part of the SEM are presented in Table 4. Negative and statistically significant relationships were found between the health facility (the facility latent variable) and delivery care quality at both admission and predelivery stages (*β* =  − 0.66, *p* < 0.001, and *β* =  − 0.73, *p* < 0.001). On the other hand, the facility latent variable was not found to significantly predict the latent for quality of postpartum delivery care (Soon_after_birth, *β* = 0.05, *p* = 0.55). Health providers’ characteristics (the provider latent variable) were also associated with delivery care quality at the first two pause points (Admission, *β* = 0.11, *p* < 0.001, and Before_Pushing, *β* =  − 0.10, *p* < 0.01). Along the care continuum, care quality at the admission (Admission, *β* = 0.48, *p* < 0.001) and predelivery (Admission, *β* = 0.29, *p* < 0.001) stages had positive and statistically significant associations with care quality at the immediate postpartum stage. Care quality at initial assessment, in contrast, had a negative and statistically nonsignificant association (Admission, *β* =  − 0.07, *p* = 0.18) with predelivery care quality.

**Table 4:** Standardized estimates of the paths relationships of the quality of care along the continuum

	Standardized coefficient (std error)	t-value
**PATHS**		
**Provider —— > Admission**	**0.11 (0.04)**	3.58 ***
**Provider —— > Before_pushing**	**−0.10 (0.04)**	−2.89 **
Provider —— > Soon_after_birth	0.05 (0.03)	1.40
**Facility —— > Admission**	**−0.66 (0.03)**	−21.40 ***
**Facility —— > Before_pushing**	**−0.73 (0.06)**	−11.70 ***
Facility —— > Soon_after_birth	0.05 (0.09)	0.59
Admission —— > Before_pushing	−0.07 (0.05)	−1.33
**Admission —— > Soon_after_birth**	**0.48 (0.06)**	3.48 ***
**Before_Pushing —— > Soon_after_birth**	**0.29 (0.08)**	4.58 ***

## Discussion

In this study, we report on the determinants of delivery care quality along the continuum of childbirth care by using different analytical approaches. Both approaches revealed that health facility and health care provider characteristics are important determinants of delivery care quality and suggest that they may have varying effects on delivery care quality at different time points in the continuum of delivery care.

All women and their newborns should receive adequate care during the intrapartum and immediate postpartum periods to advert perinatal mortality. With the increased recognition that health facility utilization for delivery does not always equate with receipt of quality care [9], there are increasing initiatives aiming to measure the care quality received by the mother–newborn dyad during the course of in-facility childbirths and its determinants [34, 35, 38–40]. Because care quality has several dimensions, studies have used different approaches and quality outcomes, including facility readiness, care outcomes and user satisfaction. More studies using process indicators are still needed, as they directly relate to the care process and are more likely to provide insights into the areas needing improvement [19, 41]. Because obstetric complications are unpredictable and occur in a relatively small proportion of women, routine care quality does matter. Routine care is what is received by the vast majority of women and their newborns, and its quality is critical for preventing complications [42]. In our results, care quality at both stages tended to be lower for nurses and auxiliary staff than for midwives. The results were, however, not statistically significant in Côte d’Ivoire. Similar results have been reported previously and may reflect differences in midwifery skills between midwives and other cadres of health workers who are not in first intention trained to provide childbirth care [23, 40]. The results were not significant in Côte d’Ivoire probably because of the distribution of the provider qualification variable in our data (approximately 90% of providers were midwives), which may have hampered investigation of its full effects on care quality. Immediate postpartum care quality significantly increased with the provider’s years of working experience in the Côte d’Ivoire data. More experienced health workers are also more likely to have benefited from quality improvement initiatives (in-service trainings, supportive supervision, etc.) that have reinforced their skills and competences [36]. Worthy of note were the results on the facility type in our study. While predelivery care quality was significantly higher in referral hospitals than in primary health care facilities in both countries, there was either no difference in the quality of initial assessment and immediate postpartum care or the difference favored primary health care facilities (in the case of postpartum care in Côte d’Ivoire). The difference in performance at the different stages between referral and primary health facilities probably reflects the difference in inputs used to define quality care at different stages. Indeed, the quality indicators at the predelivery stage are more focused on the ready availability of childbirth consumables for use by health care providers, while the indicators at the initial examination and postpartum stages are more focused on the assessment of the actual performance of clinical actions or the quality of interpersonal communication. The SEM analysis showed that while health facility and provider characteristics were significant predictors of care quality at both the admission and prepushing stages, they were not associated with immediate postpartum care quality, which was positively correlated with quality at both the admission and prepushing stages. In both countries, quality improvement strategies have recently emphasized the training of maternity care providers. The strong and significant correlations between health facilities and childbirth care quality at the admission and predelivery stages in our findings underscore the importance of an enabling environment for care quality and suggest that quality improvement interventions should not only target health care providers. The relationship between care quality at the predelivery stage and at the immediate postpartum stage was positive and significant, as was reported in a previous similar study [23]. Despite the difference between the quality inputs for the different stages, these inputs are provided by the same teams of health workers working in the same working environments, making the correlation between quality at a given phase and at the next phase obvious and relevant to account for in the regression models [43].

### Study strengths and limitations

The assessment of care quality in our study was based on a list of process indicators that have been validated for settings similar to ours. We used different analytical approaches that have the advantage, among others, of acknowledging the multidimensionality of care quality by allowing it to vary across different points in time along the care continuum instead of treating quality as a unique score with no distinction across the initial examination, predelivery and immediate postpartum periods [38]. The quality determinants were investigated for each period separately and together for all periods.

Because of the direct observation of the practices, we cannot rule out the Hawthorne effect; that is, despite the implementation of mitigating measures, some providers might have modified their practices under observation. Data were collected by eight data collectors in each country and are thus subject to an interrater reliability bias even though they were trained to standardize the processes. Our results on the quality of delivery care are constrained by the limitations of the quality index used. The focus in the design of this index was on routine care, and any extrapolation to other types of care, for example, may be less valid. The index provides a minimum set of indicators sufficient to measure delivery care quality. Other relevant quality indicators may not be included. Finally, our study did not include data on some quality improvement initiatives found relevant in previous studies: namely, the existence of quality improvement guidelines in the health facility, in-service trainings of providers and receipt of supportive supervision.

## Conclusion

Ensuring that all women and newborns have access to quality care along the continuum of childbirth care is critical for ending preventable perinatal deaths. Our findings suggest that both facility- and provider-level quality improvement strategies are needed to impact the whole continuum of delivery care. Stage-specific inputs are needed to improve delivery care quality along the continuum, and delivery care quality at an earlier stage is correlated with quality at a subsequent stage. There is a need for multifaceted interventions accounting for determinants at both the health facility and provider levels while recognizing the specific inputs relevant to each stage of delivery care.

## Data Availability

The datasets used in the current study are available from the corresponding author on reasonable request.

## Abbreviations

AMSTL: Active management of the third stage of labor
ANC: Antenatal care visit
EBP: Essential best practice
EmONC: Emergency obstetric and newborn care
HRP: Human reproduction program
ICMJE: International Committee of Medical Journal Editors
IRR: Incidence rate ratio
LMIC: Low- to middle-income countries
PHCF: Primary health care facility
SCC: Safe Childbirth Checklist
SDG: Sustainable Development Goals
SEM: Structural equation modeling
SSA: Sub-Saharan Africa
RMSEA: Root mean square error of approximation
UN: United Nations
WHO: World Health Organization
